# Assessment of the extent and monetary loss in the selected public hospitals in Jimma Zone, Ethiopia: expired medicine perspectives

**DOI:** 10.3389/fmed.2024.1283070

**Published:** 2024-02-15

**Authors:** Habtamu Getahun, Sileshi Belew, Gemmechu Hasen, Yesuneh Tefera Mekasha, Sultan Suleman

**Affiliations:** ^1^Tullu Bolo General Hospital, Oromia Regional Health Bureau, Addis Ababa, Oromia, Ethiopia; ^2^Jimma University Laboratory of Drug Quality (JuLaDQ) and School of Pharmacy, Institute of Health, Jimma University, Jimma, Oromia, Ethiopia; ^3^Veterinary Pharmacy, Pharmaceutical Quality Assurance and Regulatory Affairs, University of Gondar, Gondar, Amhara, Ethiopia

**Keywords:** cross-sectional study, wastage rate, monetary loss, expired medicines, hospitals

## Abstract

**Introduction:**

Medicine plays a crucial role in the field of healthcare as a therapeutically significant pharmaceutical product. By effectively preventing diseases, medicine has the power to save countless lives and improve the quality of life for people worldwide. However, despite hospitals' efforts to provide medical care to patients, a significant issue arises from the substantial amount of drugs that go unused due to expiration dates. This problem is particularly prevalent in resource-limited countries like Ethiopia, where the pharmaceutical supply system fails to adequately address the issue of expired drugs in public hospitals, leading to an unsatisfactory situation. Hence, the objective of this study was to assess the economic impact and volume of expired medicines in the selected public hospitals in Jimma Zone, Southwestern Ethiopia.

**Methods:**

A hospital-based cross-sectional study design was conducted to assess the economic impact and volume of expired medicines available in the public hospitals in Jimma Zone. All available hospitals that fulfilled the EFDA guidelines were included. The medication expiration rate was calculated by dividing the total monetary value of expired medicines in a year by the total value of medicines received in the same year multiplied by 100. Then, the collected data was cleared, filtered, coded, and quantitatively analyzed using the Microsoft Excel 2010 version.

**Results:**

The average medicine waste rate was 4.87% in the fiscal year of 2019/2020 and 2020/2021 in Jimma Zone public hospitals worth 32,453.3 US$. Additionally, the facility wasted an estimated of 2711.44 US$ on the disposal of expired medicines. The expiration of medicines has been linked to several issues, including near-expiry, irrational prescribing practices, and weak participation of clinicians in medicine selection and quantification of the facility. Additionally, only two hospitals had relatively good storage and handling practices.

**Conclusion:**

Overall, the expiration rate of medicines in the public hospitals in Jimma Zone was greater than the allowed level of 2%. In order to optimize the allocation of healthcare funds and ensure the appropriate use of pharmacologically significant medications it is vital to conduct a comprehensive examination at the national level within a regional hospitals.

## Introduction

Medicines are substances intended for use in the prevention, diagnosis, cure, mitigation, and treatment of disease (1). They are utilized in healthcare facilities to save millions of lives by preventing and treating diseases and to improve the human population's quality of life (2, 3). In the process of ensuring the availability of medicines to the population, particularly in public hospitals, there is a possibility that these medicines may be damaged or expired and become unsafe for use (4). For instance, it's crucial to be aware of the finished pharmaceutical product's typical expiration date. Zilker et al.'s systematic evaluation from 2019 found that the active pharmaceutical ingredient (API) content had to fall between 95 and 105% of the specified range for the entire time it was on the market (5). The finished pharmaceutical items are given a shelf life based on the outcomes of real-time and accelerated stability tests. The study showed that the average expiration period of medications is typically set in the range of 1–5 years (6). Manufacturers typically estimate expiration dates conservatively because conducting such thorough investigations is expensive and time-consuming. For hospitals, nursing homes, and organizations that stockpile significant quantities of medications, such as the German armed forces (Bundeswehr) and the US Department of Defense, short expiry dates of medical items present a financial obstacle (6). For instance, hospitals are an area where a great number of patients were admitted during the era of COVID-19 (7, 8). Additionally, a high amount of financial resources were spent on the health sector, which was estimated at 18.2 billion Ethiopian birr in 2015/16, and 21.7 billion Ethiopian birr, respectively (9).

Even with the high amount of money and the possibility of expired medicines found in the health sector, minimum attention is given to pharmaceuticals when compared with the public health impact (10). As evidenced, the gross domestic product of Ethiopia funded by medicinal and pharmaceutical products is estimated to be 1,765.335 US$ per year from 1995 to 2022 (11). According to a study, despite hospitals being sites where patients receive medical treatment, the projected total value of medications withdrawn owing to expiration dates in 2010 equaled 0.045% of the money spent on the acquisition of all drugs in the Clinical Center of Serbia (12). This revealed that hospital systems to combat expired medicine expenditures were limited. Manufacturing companies all around the world produced a large quantity of pharmaceuticals to ensure that the general population had access to medication (13). However, little emphasis was paid to combating expired medication in health facilities. The World Health Organization confirmed that the probability of detecting pharmaceutical or medical waste as “expired, and unused pharmaceutical products, drugs, vaccines, and sera in health institutions” exists (14).

Pharmaceutical wastage is caused by different factors like inadequate inventory management, long procurement processes, poor storage, inappropriate monitoring of drug expiry times, distribution issues, and irrational drug use (15). This led to a waste of financial resources, a lack of vital drugs, and a reduction in healthcare service quality (16). In developing countries, medicine waste due to expiration is a major problem that leads to economic losses. The national stores and public-sector health institutions in low- and middle-income countries like Uganda have large stockpiles of medicines that have passed their expiration dates (17, 18).

Pharmaceutical waste is a substantial source of economic loss. Such an impact is expected to be high in developing nations, given the budget constraints in their healthcare systems (17). A report from South Africa indicated that an estimated $66,000 worth of drugs had expired (19). From an Ethiopian legal framework standpoint, Suleman et al. confirmed that the existence of legislation alone does not guarantee the absence of unauthorized or illegal medicine sources, as effective implementation of the legislation is affected by the government's political commitment, resources, and intergovernmental cooperation (20). Furthermore, the country's pharmaceutical supply chain agency is not entirely robust in the regulation of medicine distribution to target sites, causing drug distribution to exceed the requirements of health facilities (21–23). This was proved by long delivery times, frequent stockouts of essential medicines, inadequate skilled human power, an intermittent and inaccurate reporting system, and a lack of top-level management commitment, which in turn led to inflated product costs, a low rate of delivery of goods, and the flexibility of the organization (24).

According to the literature, inventory management practices in Ethiopia resulted in ~2.1% of medications being wasted in 1 year owing to expiration and damage (25). This resulted in a loss of more than $2 million. Another study conducted in Amhara regional state health facilities found that an average of 1.1% wastage rate was reported in auditable pharmaceutical and transaction systems implementing hospitals (26).

Wastage of medicines through expiration is becoming a serious problem for the government in Ethiopia due to the financial crisis. A study in Ethiopia highlighted the extent of medicine waste in health facilities, and none of the studies reviewed looked into the pattern of pharmaceutical categories of wasted medicines. As evidenced, the study conducted in six facilities in Gondar town revealed that about 26,760 ETB were lost over 6 months due to expiration (27). Additionally, the study conducted from 29 March to 29 April 2016 in public health facilities in East Shewa Zone, Oromia Regional State, revealed that a total of 174,366.98 Ethiopian Birr was lost in health facilities because of expired medicines (28).

Ethiopia faces a significant challenge in effectively managing its medical supply chain, particularly when it comes to drug waste. The Health Sector Development Program (HSTP II) has set a national goal of reducing the wastage rate of medicines to < 2% (29). In Ethiopia, similar to other developing nations, it is imperative to closely monitor a health supply chain issue arising from drug wastage (30). During the assessment conducted in 2013/2014 while gathering baseline data for Auditable Pharmaceutical Transaction and Services (APTS) in government hospitals of the Federal and Addis Ababa City administrations, it was found that the average medicine wastage rate over a span of 3 years (2012, 2013, and 2014) in eight hospitals was 4.8%. This amounted to a total of 11,078,910.52 Ethiopian Birr (ETB) (29).

In the south-west districts, especially the Jimma Zone, where a huge pharmaceutical transaction was exchanged and high demand of business swapped targeted pharmaceutical products. Ethiopian hospitals' reform implementation was launched a guideline in 2010 regarding supply chain in hospitals. The reform recommended that guidelines be introduced with the aim of ensuring uninterrupted supply of quality, and cost-effective pharmaceuticals at all public hospitals, and these measures are also required to combat expired medicines (31). By considering the above pharmaceutical issues, the present study aimed to assess the extent and monetary loss due to expired medicines in the selected public hospitals in Jimma Zone, Ethiopia.

## Materials and methods

### Study setting

The study was conducted in Jimma Zone public hospitals. The zone is included in the south-west district of Oromia Regional State, Ethiopia. The expired medicines were collected from 2019/2020 to 2020/2021 that were expired and stored in the selected hospitals found in the study sites. The town is located 355 km away from the capital city of the country, Addis Ababa. According to the Jimma Zone Health Bureau reports, Jimma Zone has community pharmacies (*n* = 26), drug stores (*n* = 103), 11 hospitals (general hospitals = 3, referral hospitals = 1, and primary hospitals = 7), health centers (*n* = 126), and health posts (*n* = 555) that provide health services for the total population of 2,986,957, according to the 2017 Central Statistical Agency report (32).

### Study design and research questions

A hospital-based cross-sectional study design was conducted to assess the economic impact of expired medicines in the public hospitals in Jimma Zone. The collected data from expired medicines were described quantitatively to estimate the extent and monetary loss due to expired medicines in the selected area. The 2-year financial loss was estimated from expired medicines. The research methodology was driven by the basic research question of (1) how much of the expired medicines are available in the health facilities and (2) what is the total cost of those expired medicines in the two fiscal years (2019/2020 and 2020/2021)? The research questions were answered through five data collection tools, which describe the expired medicines data collection (Supplementary material 1), the checklist on procedures for expired medicines storage and handling practice (Supplementary material 2), questionnaires on storage and disposal practice of expired medication (Supplementary material 3), questionnaires on storage and disposal practice of expired medication (Supplementary material 4), and perceived factors associated with medicines expiry at the public health facility (Supplementary material 5).

#### Source and study population

All of the public hospitals found (*n* = 11) in Jimma Zone, Ethiopia, were used as a source of population. However, only nine hospitals (9/11) were involved in the study by considering adequate records of expired medicines. The three hospitals were not included because of inadequate information concerning expired medicines.

### Sampling size determination and sampling technique

The sample size was determined by considering the availability of the hospitals found in the Jimma Zone that provide health services. By taking into account the Food, Medicine, and Healthcare Administration and Control Authority guidelines, only nine hospitals (*n* = 9) were selected for this study from among the available hospitals. They provide a wide range of services, important information, and adequate records on expired drugs. Ethiopian Food, Medicine, and Healthcare Administration and Control Authority recommends that the health sector have critical medical information, an adequate unused drug record system, and provide a wide range of services for the whole public, taking into account any scientific findings and drawing possible solutions for the pharmaceutical situation that happened in the system (33). There was no sampling technique employed to choose the health facilities. All hospitals in the zone were included. The extent and monetary value were determined from expired medicines recorded with prices from the two fiscal years (2019/2020 and 2020/2021). The pharmacy store manager and pharmacy head were purposefully selected regarding factors contributing to the expiration of medicines in the hospitals because they are supposed to be more information-rich than other health professionals. The quantitative data were collected using a standard checklist through observation, document review from the literature, and the expired medicines register book. The closed-ended and self-administered questionnaires were employed to collect information on expired medicines in their respective facilities.

### Inclusion and exclusion criteria

#### Inclusion criteria

The nine selected hospitals (*n* = 9) found in the Jimma Zone that are active and fulfill the Food, Medicine, and Healthcare Administration and Control Authority guidelines were included. Additionally, volunteer public facilities with records of expired medicines and data on expired medicines for at least 1 year prior to the collecting period were included. All pharmacy professionals who were present at the study site and volunteered to participate in the study during the study period were included, as were all expired medicines recorded with prices in the previous two fiscal years (2019/2020 and 2020/2021).

#### Exclusion criteria

The public facilities that had inadequate data records, expired medicines from the program, and anticancer drugs were excluded for volume and cost analysis. Furthermore, expired supplies and laboratory commodities were excluded from the estimation of the extent, and cost assessments were also excluded.

### Data collection tools

Previously published literature and guidelines were used for the quantitative study to review all records of the expired medicine file and to abstract secondary data on the extent and types of expired medicines, cost loss, and disposal practice. Expired medicines found at each hospital were studied for their type, unit, dosage forms, cost, and quantity using the adopted format from Ethiopian pharmaceutical disposal rules. The questionnaires used for assessing factors associated with drug expiry in the hospitals that were distributed to the store manager and pharmacy head were adopted with some modifications from the previously published peer-reviewed journals (33–37).

The five-point Likert scale was employed for assessing factors associated with the expiration of medicines in the studied health facilities. The facility was assessed through a total of 19 questionnaires prepared as per a Likert scale that ranged from strongly disagree (*n* = 1) to strongly agree (*n* = 5). The observational checklist and close-ended questionnaire were adopted using Ethiopian pharmaceutical disposal rules to assess the storage conditions and disposal practices of expired pharmaceuticals at the facility (33).

The storage and handling practice of expired medicines in public hospitals were settled by using 10 selection criteria (Supplementary material 2). The facility fulfillments of the storage condition: those facilities that fulfilled the criteria more than or equal to 80% (mean) were considered as good, while those fulfilled < 80% were considered as poor storage conditions (28).

### Data quality control

The study questionnaire was designed after reviewing various literature and guidelines published in English by the Pharmaceutical Supply Chain Agency and the Ethiopian Food, Medicine, and Healthcare Administration and Control Authority. Prolonged engagement with data, persistent observation, negative case analysis, and information adequacy were all strategies employed for data quality control, all of which can be utilized to boost the credibility of quantitative studies. Data from all selected hospitals were coded, reviewed for accuracy, consistency, and omissions, and then prepared for analysis using approved data collection forms. The quality of the data was checked by the research team (HG, GH, SB, YT, and SS) by providing training for 2 days for three professionals prior to embarking on the data collection process (Supplementary material 6) regarding expired medicines to give trainers information about where the data were collected, types of data included and excluded, as well as general information about the medicine's environment (name, strength, class, etc.).

The self-administered questionnaires were administered prior to collecting the data, and a pilot study was done to standardize the validity of the data collection tool. Those facilities that participated in the pilot study were excluded from the study samples. The principal investigator was assigned to supervise the data collection process, and any inconsistencies were taken into account before the next phase of sample collection started.

### Data analysis and interpretation

The collected data were entered, filtered, coded, and analyzed using the Microsoft Excel 2010 version. The economic impact of expired medicines was evaluated as per Health Economic Evaluation Reporting Standards (38). The rate of medicine wastage was determined by calculating the percentage obtained by dividing the monetary value of wasted medicines by the total value of medicines received in the same year (39, 40) using Equation 1.


(1) $$\begin{array}{c} \text{Medicine wastage rate}(\%)\\ =\frac{\text{Value of wasted medicines in a year}}{\text{Total value of medicines received during the same year}}\text{ x 100} \end{array}$$

If the rate of medicine wastage exceeds the permitted level of 2%, it indicates a higher rate of medicine wastage (29). Then descriptive statistics were generated and evaluated to provide the requisite data from the available information. The categorical data were calculated as frequencies and percentages. Finally, the results and findings were presented in graphs and tables, depending on the nature of the variables under consideration.

### Ethics statement

Ethical approval was obtained from the Ethics Review Committee of the Institute of Health, Jimma University (JHRGn/248/21) and the Jimma Zone Health Bureau. The study was then carried out following approval from each individual public health facility. The goal of the study was explained to the study participants, and their verbal informed consent was obtained. The ethics review committee authorized verbal informed permission for this investigation, and the confidentiality of study participants' data was upheld throughout.

## Results

### Volume and cost loss of expired medicines

By considering the adequacy of expired medicine data, nine hospitals from the 11 found in the Jimma Zone were included in the study. The data of 2 years, from 2019/2020 to 2020/2021, of expired medicines from manual and electronic records were reviewed, and pharmaceutical logistic data were collected. The result showed that 32,453.3 US$ were lost in value in the studied public hospitals due to the expiration of medicines.

As can be revealed in Table 1, without the involvement of Hospital-3, about 4.86% of losses were investigated due to expired medicines in the year 2019/2020. On the other hand, about 4.87% of losses were recorded due to expired medicines found in nine hospitals (*n* = 9) in the years 2019/2020 to 2021. The overall aggregated mean wastage rate of expired medicines in the study hospitals was ~4.87% in the two fiscal years. The volumes of expired medicines per public hospital are presented in Table 1.

**Table 1:** Volume of expired medicines in the facilities by year.

	**2019/2020**			**2020/2021**		
**Hospital**	**Received budget (US$)**	**Expired in monetary (US$)**	**%**	**Received budget (US$)**	**Expired in monetary (US$)**	**%**
Hospital-1	35,398.59	1,190.351772	3.4	33,430.7021	536.0638106	1.6
Hospital-2	11,378.75	632.1158713	5.6	17,201.10864	483.9685466	2.8
Hospital-3	—	—	—	13,885.51609	850.3844902	6.1
Hospital-4	165,352.89	8,730.860629	5.3	170,550.6898	11,053.94794	6.5
Hospital-5	23,302.73	810.3693059	3.5	27,355.30405	1,066.693601	3.9
Hospital-6	16,978.18	696.760846	4.1	24,535.01392	902.3994939	3.7
Hospital-7	22,405.68	1,337.69107	5.9	27,714.88359	517.0412148	1.9
Hospital-8	10,041.69	342.1006869	3.4	19,630.47831	467.3107375	2.4
Hospital-9	25,897.54	1,377.148771	5.3	21,828.62997	1,458.181309	6.7
Total	310,756.05	15,117.4	4.86%	356,132.3	17,335.9	4.87%

1 US$ = 55.32 Birr, an average conversion rate has been used (22 October 2023).

**Figure 1:**
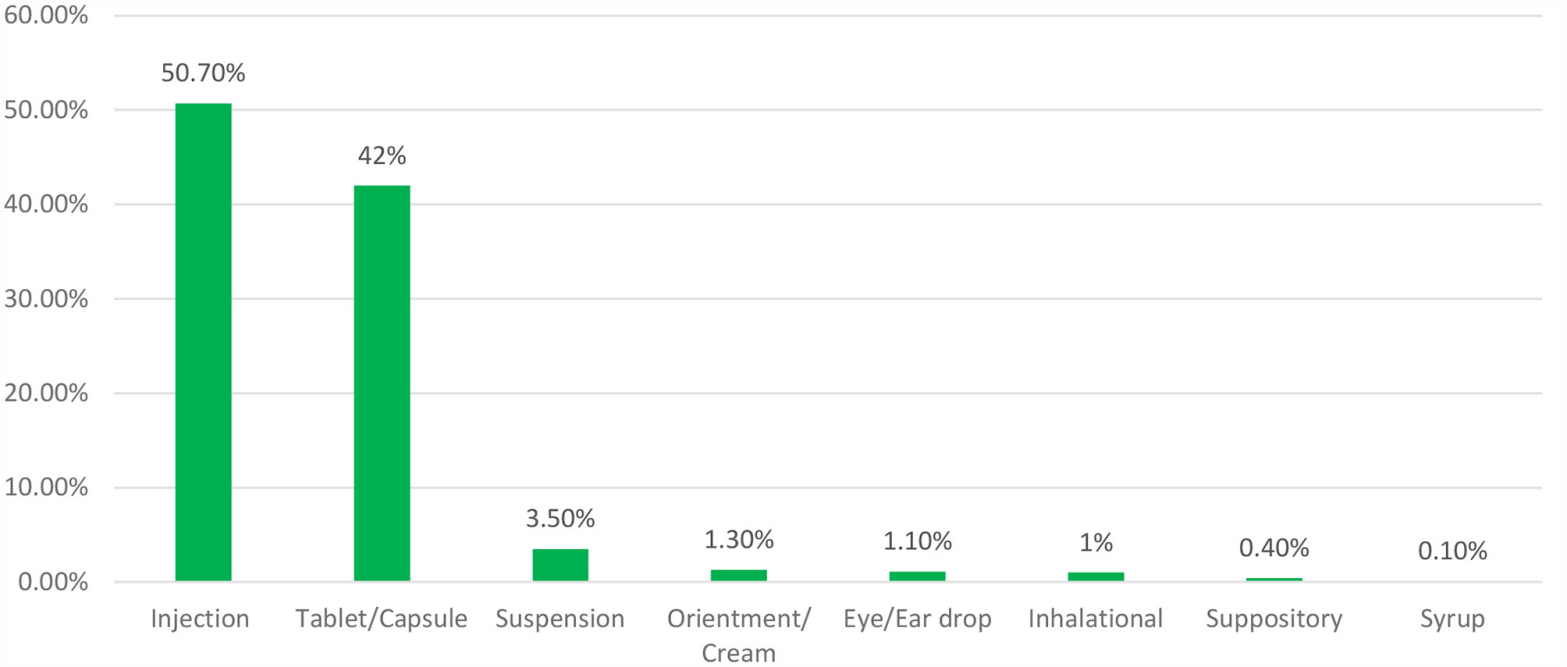
Types of expired drugs based on their route of administration.

Overall, 478 dosage forms were found to be recorded in the assessed public health facilities. Solid dosage forms (51.99%) and liquid dosage forms (45.34%) were the most commonly expired medicines in the evaluated hospitals with a significant budget. Semisolid dosage forms (1.72%) and inhalational dosage forms (0.95%) were the least exposed to expiration in public hospitals. From the types of dosage forms based on route of administration, an estimated 50.7% of injectable dosages were lost, followed by tablets as well as capsules, which both accounted for 42% of losses in the respective hospitals (Figure 1).

**Figure 2:**
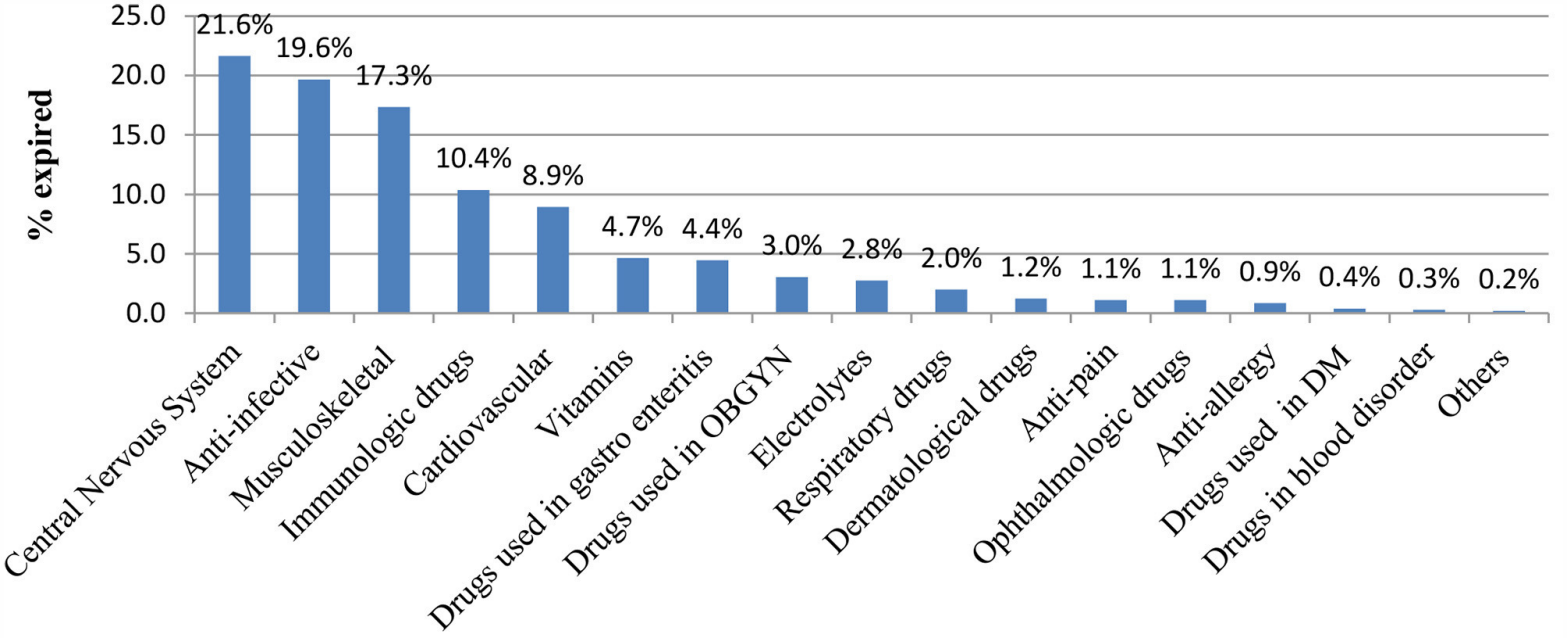
Expired medicines based on their ATC classifications.

From the therapeutic class of drugs, the central nervous system and anti-infective drugs were the highest expired classes of drugs in the hospitals, accounting for 21.6 and 19.6%, respectively. The overall expired medicines according to the anatomical therapeutic class are depicted in Figure 2.

**Figure 3:**
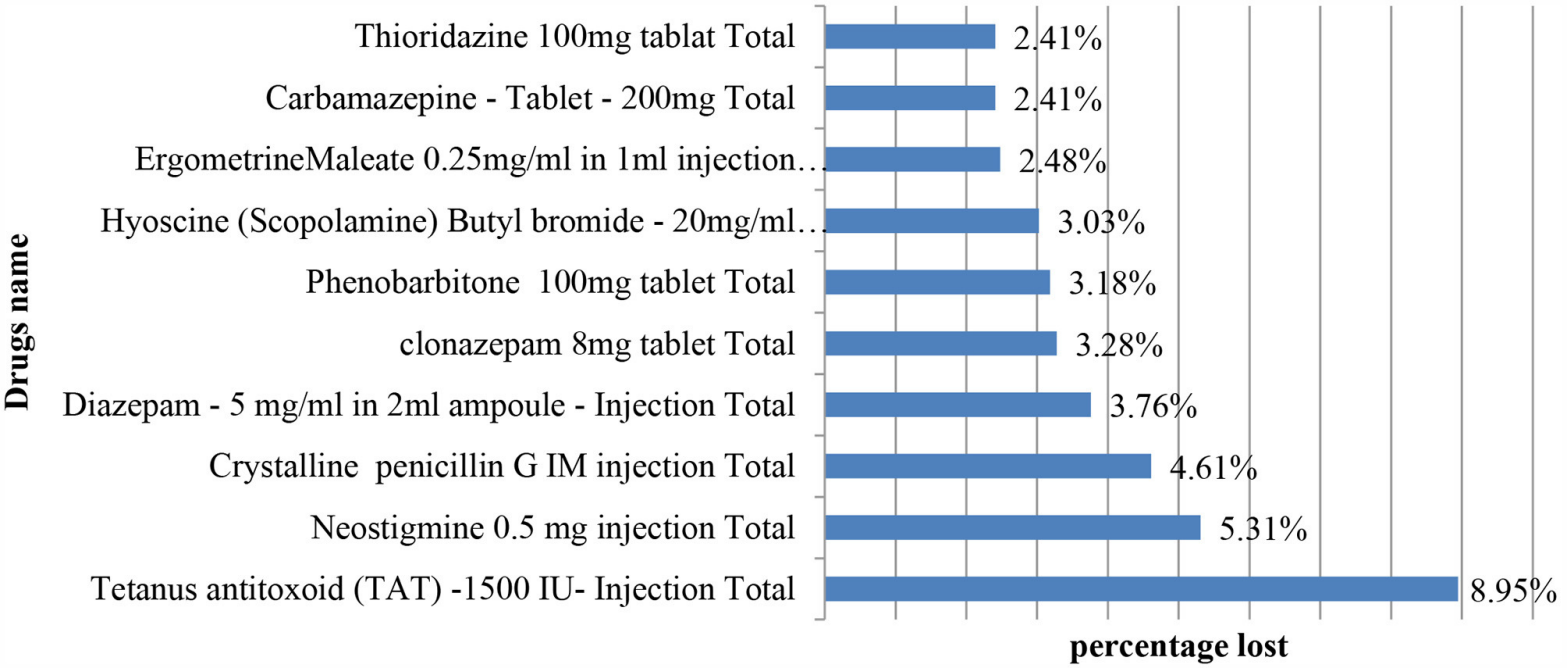
Top 10 expired medicines in the assessed facilities.

From a single form of drug point of view (Figure 3), tetanus-antitoxin injection (8.95%), neostigmine 0.5 mg/ml injection (5.31%), and crystalline penicillin G injection (4.61%) were the three most expired drugs in the facilities, respectively (Figure 3).

### Factors associated with expiry of medicines

**Figure 4:**
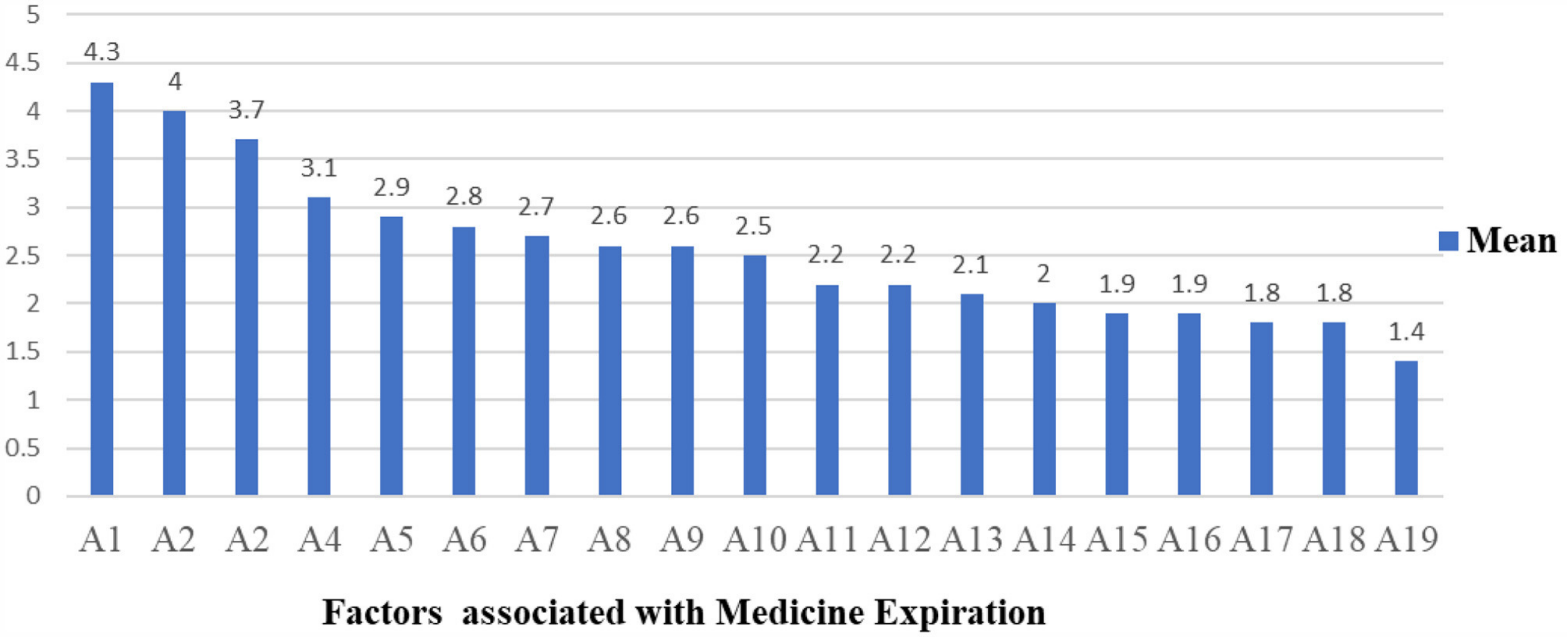
The mean of factors associated with medicine expiration in the study area. A1, A2, A3, A4, A5, A6, A7, A8, A9, A10, A12, A13, A14, A15, A16, A17, A18, and A19 stand for factors associated with the expiry of medicines in the studied health facilities that are described in Table 2, respectively.

The hospitals were assessed as per a Likert scale that ranged from strongly disagree (*n* = 1) to strongly agree (*n* = 5). A total of 19 questionnaires were distributed to 18 respondents and received a 100% response rate. Two study participants (1 = store manager and 1 = pharmacy head) were selected from each of the nine hospitals that represented the staff who work in the health facilities. Near-expiry and irrational prescriptions were found to be the most associated factors with medicines expiring in public health facilities, with an average response of 4.3 and 4, respectively. Another 3.7% of average respondents agreed that clinicians' weak involvement in the selection and procurement of pharmaceuticals was among the factors leading to medicine expiry in the public health facilities studied (Figure 4 and Table 2). The general description of the factors that are likely associated with medicine expiration in public hospitals is described in Table 2.

**Table 2:** Factors associated with the expiry of medicines in the studied health facilities.

**S. No**.	**Associated factors**	**NR**	**SDA**	**DA**	**N**	**A**	**SA**	**Mean**
			***n*** **(%)**	***n*** **(%)**	***n*** **(%)**	***n*** **(%)**	***n*** **(%)**	
A1	Near-expiry medicines (< 6 months) are being delivered to the health facility	18	0 (0)	0 (0)	0 (0)	13 (72.22)	5 (27.78)	4.3
A2	Irrational prescribing causes underuse of certain medicines	18	0 (0)	0 (0)	0 (0)	18 (100.00)	0 (0)	4.00
A3	Weak participation of clinicians in medicine selection and quantification	18	0 (0)	3 (16.67)	0 (0)	14 (77.78)	1 (5.56)	0.7
A4	Lack of electronic stock management tools in the health facility	18	0 (0)	9 (50.00)	1 (5.56)	6 (33.33)	2 (11.11)	3.1
A5	Weak functional DTC in the health facility	18	3 (16.67)	5 (27.78)	0 (0)	10 (55.56)	0 (0)	2.9
A6	No accurate data available in the health facility to facilitate quantification	18	1 (5.56)	7 (38.89)	5 (27.78)	5 (27.78)	0 (0)	2.8
A7	The presence of overstocked medicines due to poor quantification	18	1 (5.56)	10 (55.56)	1 (5.56)	5 (27.78)	1 (5.56)	2.7
A8	Weak or no mechanisms for medicine expiry monitoring and evaluation	18	2 (11.11)	9 (50.00)	2 (11.11)	4 (22.22)	1 (5.56)	2.6
A9	Minimum shelf life is not specified in orders	18	3 (16.67)	8 (44.44)	1 (5.56)	6 (33.33)	0 (0)	2.6
A10	The shortage of pharmacy human resources in the facility	18	2 (11.11)	8 (44.44)	5 (27.78)	3 (16.67)	0 (0)	2.50
A11	No timetable for regular inventory level analysis	18	3 (16.67)	11 (61.11)	2 (11.11)	1 (5.56)	1 (5.56)	2.2
A12	Lack of system to move nearly expired medicines from facility to facility	18	3 (16.67)	11 (61.11)	1 (5.56)	3 (16.67)	0 (0)	2.2
A13	Expired medicines not isolated into secure areas	18	1 (5.56)	15 (83.33)	1 (5.56)	1 (5.56)	0 (0)	2.1
A14	Medicines are purchased without procurement plan/policy in the facility	18	5 (27.78)	11 (61.11)	0 (0)	1 (5.56)	1 (5.56)	2
A15	Medicines are not arranged systematically on shelves in the facility store	18	3 (16.67)	14 (77.78)	1 (5.56)	0 (0)	0 (0)	1.9
A16	Poor stock management like using neither FIFO nor FEFO in stock	18	2 (11.11)	16 (88.89)	0 (0)	0 (0)	0 (0)	1.9
A17	Selection of medicines is not based on the available essential medicines list	18	3 (16.67)	15 (83.33)	0 (0)	0 (0)	0 (0)	1.8
A18	Abrupt changes in treatment practices result in medicine expiration	18	3 (16.67)	15 (83.33)	0 (0)	0 (0)	0 (0)	1.8
A19	Lack of accountability for the expiry of medicines in the facility	18	12 (66.67)	5 (27.78)	0 (0)	1 (5.56)	0 (0)	1.4

NR, number of responses; SDA, strongly disagree (1); DA, disagree (2); N, neutral (3); A, agree (4); SA, strongly agree (5).

### Storage and handling practice of expired medicines in the public hospitals

**Figure 5:**
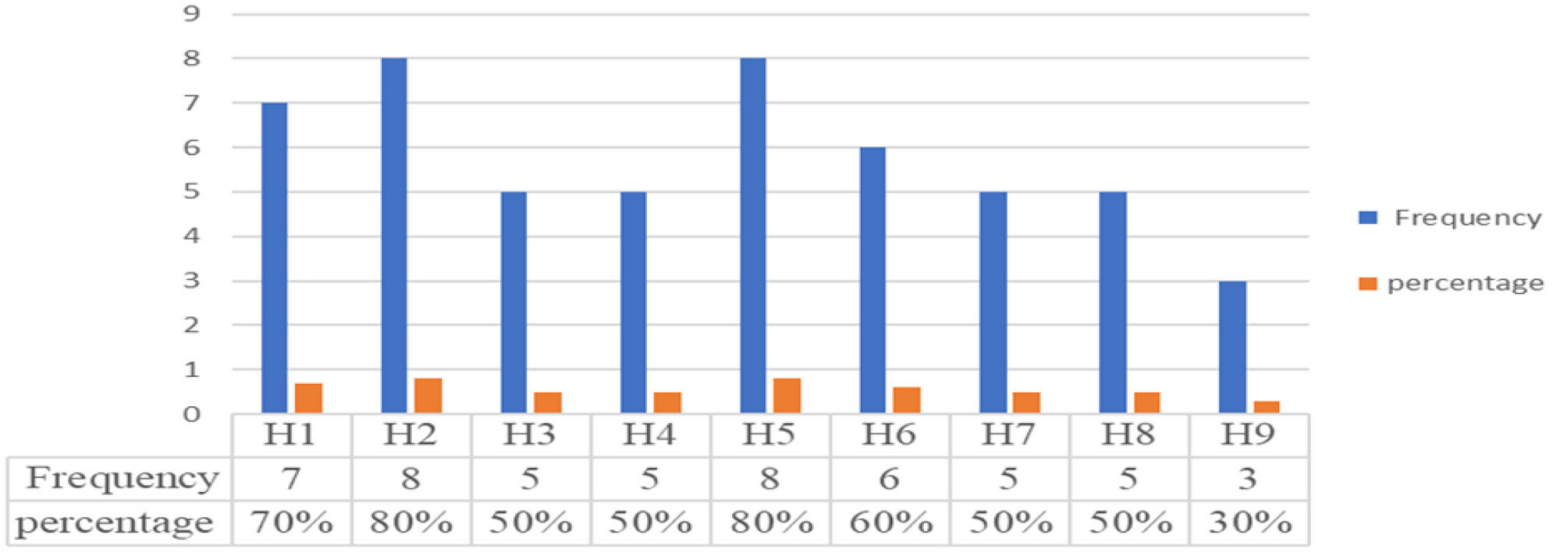
Handling and storage practice of expired medicines in the studied hospitals. H1, H2, H4, H5, H6, H7, H8, and H9 stand for list of hospitals.

Depending on the selected 10 criteria, the storage and handling practices of expired medicines were assessed through an observational checklist. The facility fulfillment of the storage condition: those facilities that fulfilled the criteria more than or equal to 80% were considered as good, while those fulfilled < 80% were considered as poor storage and handling practice conditions (29, 31, 41). Accordingly, hospitals that scored greater than or equal to the average result (≥80%) were assigned as good, and those that scored less than the average (< 80%) results were assigned as poor storage and handling practices. The lack of labeling of storage space and the storage of drugs without regard to their pharmacological groups (Table 3) were the two common problems encountered in all assessed hospitals. Additionally, poor management was evident in some public hospitals in the separation of expired medicines depending on their disposal method due to a lack of storage space. The findings of all elements used to evaluate the storage and handling practices of expired medicines in the selected hospitals of the Jimma Zone are described in Table 3. As can be seen in Figure 5, Hospital-5 and Hospital-2 fulfilled the eight (8/10) criteria for the storage and handling practice of expired medicines. This suggests that only two hospitals had relatively good storage and handling practices for expired medicines (80%). The remaining seven hospitals deviated from storage and handling compliance by scoring < 60%.

**Table 3:** Handling and storage practice of expired medicines in public hospitals.

**Storage practices of expired medicines**	**List of hospitals**								
	**Hosp-1**	**Hosp-2**	**Hosp-3**	**Hosp-4**	**Hosp-5**	**Hosp-6**	**Hosp-7**	**Hosp-8**	**Hosp-9**
Maintained register	Yes	Yes	Yes	Yes	Yes	Yes	Yes	Yes	Yes
Availability of past disposal application form	Yes	Yes	No	No	Yes	Yes	Yes	No	No
Segregated from usable medicines	Yes	Yes	Yes	Yes	Yes	Yes	Yes	Yes	Yes
Segregated based on their disposal method	No	Yes	No	Yes	Yes	No	No	Yes	No
Separate storage area	Yes	Yes	Yes	Yes	Yes	Yes	Yes	Yes	Yes
Storage area labeled properly	No	No	No	No	No	No	No	No	No
Enough storage area	Yes	Yes	Yes	No	Yes	Yes	No	No	No
The presence of adequate security measures	Yes	Yes	Yes	Yes	Yes	Yes	No	Yes	No
The presence of previous disposal records	Yes	Yes	No	No	Yes	No	Yes	No	No
Segregated based on their pharmacological groups	No	No	No	No	No	No	No	No	No

1: The presence of handling practice (yes), the absence of handling practice (no).

Hosp, hospital.

### Disposal practices of expired medicines in public hospitals

In the study, except for one hospital, all of the public hospitals investigated have a history of disposing of pharmaceuticals (Table 4). All of them obtained approval from the appropriate regulatory agency before disposing of expired medicines. The minimum year for storing expired medicines in the facility was found to be one and a maximum of 5 years. Only one hospital disposed of expired medicines every year, while three others disposed of those medicines stored for a minimum of 2 years at once, and five assessed hospitals still have expired medicines that have passed their expiration date by a minimum of 1 year and a maximum of 5 years. In addition to the value lost as a result of expired drugs, the facility wasted an estimated 2,711.49 US$ for the disposal of expired medicines. Open burning and landfills were the two most regularly used methods for disposing of expired medicines. A shortage of storage space was the most commonly reported concern arising from the long-term storage of expired medicines in public health facilities. The most commonly reported barrier to disposing of expired pharmaceuticals in public hospitals was a lack of disposal facilities (Table 4).

**Table 4:** Maximum years of expired medicines stored in assessed public hospitals and associated cost loss due to disposal.

**List of hospitals**	**Maximum year of medicines stored prior to disposal**	**Methods frequently reported**	**Disposal year**	**Estimated amount of Birr wasted per disposal (US$)**
Hospital-1	2	Landfill, open burnt	2013 E.C	180.77
Hospital-2	2	Landfill, open burnt	2013 E.C	271.15
Hospital-3	1	Landfill, open burnt	—^*^	—
Hospital-4	5	Landfill, open burnt	Before 5 years	542.29
Hospital-5	1	Landfill, open burnt	2013 E.C	361.53
Hospital-6	2	Landfill, open burnt	Before 2 years	180.76
Hospital-7	2	Landfill, open burnt	2013 E.C	361.53
Hospital-8	2	Landfill, open burnt	Before 2 years	271.14
Hospital-9	4	Landfill, open burnt	Before 4 years	542.29
Total				2,711.49

1 US$ = 55.32 Birr, an average conversion rate has been used (22 October 2023). The symbol ^*^ represents no history of disposal.

## Discussion

From an Ethiopian health facility point of view, effectively using pharmaceutical products in the health environment requires urgent action to be taken to implement a policy on pharmaceutical supply chain monitoring, which has a greater contribution to safely using health budgets in the healthcare system (42, 43). The study's findings provide solid pharmaceutical information about the extent of and monetary losses caused by expired medicine, storage and handling practices, and disposal procedures in the public hospitals in the Jimma Zone.

Medicine waste has a significant financial impact on the healthcare system as well as negative environmental consequences. In addition to this, access to quality medicines is a global public health problem, especially in resource-limited countries (44, 45). However, a large number of expired medicines are available in Ethiopian public health facilities, as they are in other developing countries around the world. Thus, evaluating the extent and associated cost of these medicines is important for further targeted interventions. In this study, a total of nine public hospitals found in the Jimma Zone of Oromia Regional State were included.

In this study, the data of 2 years, from 2019/2020 to 2020/2021, of expired medicines from manual and electronic records were reviewed, and pharmaceutical logistic data were collected. The result showed that 32,453.3 US$ were lost in value in the studied public hospitals due to the expiration of medicines. Similarly, studies from South African hospitals found that R700,000 was lost due to expired medicines (46). Also in Uganda, about $1,307 was lost only from 13 expired essential medicines (47). This study showed that financial loss due to expired medicines remains common in the studied public health facilities across African countries.

Additionally, about 4.86% of losses were investigated due to expired medicines in the year 2019/2020. On the other hand, about 4.87% of losses were recorded due to expired medicines found in nine hospitals (*n* = 9) in the years 2019/2020 to 2021. The overall wastage rate of expired medicines in the study hospitals was ~4.87% in the two fiscal years. The wastage rate of medicines must be kept below 2% in accordance with the national objective of reducing the wastage rate of medicines set by the Health Sector Development Program (HSTP II) (29). Therefore, the current investigation has indicated that the disposal rate of medications in public hospitals exceeded the permitted national waste rate of 2%.

Solid dosage forms (51.99%) and liquid dosage forms (45.34%) were the most commonly expired medicines in value found in the evaluated hospitals. Different study findings reported from the health facilities of the central Oromia region in south-west Shoa (34) and the west Wollega zone (48) revealed that the percentage wastage rate of expired medicines was high (7.5%) and 8.04% as compared to the present study, respectively. However, the findings of the current study were reported as high when compared with a study conducted in Amara region, Dessie town, Ethiopia, which reported that about 3.68% of medicine expired in the health facilities (49). This could be due to the variety of public health institutions covered, the types of drugs used, and the study period's fluctuation. This study found a relatively high percentage of drug waste in the public health institutions investigated. According to the study, from the types of dosage forms based on route of administration, an estimated 50.7% of injectable dosages were lost, followed by tablets as well as capsules, which both accounted for 42% of losses in the respective hospitals. In line with this study, an identical finding was reported from Ethiopian health facilities, which showed that tablets (20.78%) and injectables (16.49%) constituted the highest wasted dosage forms (49). From the therapeutic class of drugs, the central nervous system and anti-infective drugs were the highest expired classes of drugs in the hospitals, accounting for 21.6 and 19.6%, respectively. The presence of anti-infective drugs that are expired shows the probability of bacterial resistance if inappropriately disposed of, and central nervous system drugs may lead to reuse and misuse because they consist of narcotic drugs (50). Similarly, the study carried out at a tertiary hospital in Dar Es Salaam (51) and Awi Zone in Ethiopia (36) showed that anti-infective drugs were the most frequently expired medications, with wastage rates of 18.9 and 36.4%, respectively. The study also attempts to report the prevalence of expired medicine from a single form of drug. Accordingly, tetanus-antitoxin injection (8.95%), Neostigmine 0.5 mg/ml injection (5.31%), and crystalline penicillin G injection (4.61%) were the three most expired drugs in the facilities, respectively. Identical findings were reported from the Ethiopian Western cluster of pharmaceutical supply chains, which reported that tetanus antitoxin (TAT), in terms of single drug value, had the highest drug expiry (4,110,426.43 ETB: 20%) (52).

Nineteen self-administered questionnaires were distributed to 18 respondents in order to determine the key factors that contribute to the expiration of medicines in health facilities from the perspective of medicine exposure. In the current study, near-expiry and irrational prescriptions were found to be the most associated factors with medicine expiry in public hospitals. These situations may increase antimicrobial drug resistance. The study conducted in 2019 indicated that an estimated 1.27 million deaths were directly attributed to bacterial antimicrobial resistance (AMR), with sub-Saharan Africa having the greatest burden (53). This discovery suggests that it is crucial for healthcare professionals to receive training on rational antimicrobial prescribing or antimicrobial stewardship as a fundamental component of initiatives aimed at reducing antimicrobial resistance.

One of the issues of health supply chain management in underdeveloped nations, particularly Ethiopia, is pharmaceutical expiration. This was due to a lack of reliable data on the detailed underlying variables related to expired drugs in public hospitals. Medicine wastage not only hampers therapeutic benefits but also affects financial capability.

On average, countries spend about 25% of their total health expenditure on medicines. Of these, according to Management Science for Health estimations, 70% of the total funds invested in essential medicines are wasted in normal supply systems (54). This was addressed by identifying elements that contribute to drug expiration. According to a study, factors linked to drug waste in healthcare settings are not extensively documented in low-income countries. According to a report, the majority was attributed to inadequate supply chain management systems (55, 56).

Additionally, clinicians' weak involvement in the selection and procurement of pharmaceuticals was among the factors leading to medicine expiry in the public health facilities studied. In line with this study, a study from Uganda (56) and another study from Ethiopia (40) reported that poor procurement planning and the management of medicines near expiration were the most associated factors with the expiration of medicines. This may show that factors related to pharmaceutical expiry are a continuous problem in today's medicine supply chain in public health facilities. The study also identified a lack of electronic stock management tools in health facilities as another cause of medicine wastage. A similar study done in Ethiopia indicates a lack of electronic stock management tools that automatically capture drug information was the cause of medicine wastage in the facilities (40).

Another finding of the study revealed that inadequate stock management practices, such as the absence of both FIFO and FEFO methods, played a significant role in the occurrence of medication expiration in public health facilities. A study conducted in Ethiopia by Diriba et al. also highlighted similar results, stating that ineffective store management, policies, and standards were the primary factors leading to medicine expiration at the health facility level, as reported by key informants (52). To address the issue of medicine expiration in health facilities, it is crucial for all pharmaceutical supply chain agencies at both the federal and regional levels to prioritize and adopt strong inventory management systems. These systems will ensure the efficient supply of medicines to health facilities, thereby defending against contributing factors that lead to expiration.

The presence of overstocked medicines in the facility was also a factor contributing to the medication wastage, as per the present findings. Similarly, a study performed in Uganda (35) and Tanzania (51) showed that overstocking of medicines was one of the major contributing factors to the expiration of medicines. Overstocking of medicines normally leads to a high number of expired medicines, a high cost of storing excess stock, and high incidences of pilferage of highly potent medicines (40, 57). Consequently, this situation can lead to suboptimal utilization of consumption data and a lack of involvement from healthcare professionals in the process of forecasting and selecting medicines. Therefore, a substantial endeavor is required within public health facilities to diminish the expiration rate by enhancing the quantification and selection of medicines.

It is critical to maintain optimal storage conditions in healthcare facilities in order to decrease pharmaceutical waste caused by environmental variables. The expiration of medicines in healthcare institutions may result in the waste of potentially life-saving drugs as well as wasteful costs for the disposal of those outdated medicines (48). In the study, the handling and storage practices of the public hospitals were assessed based on an observational checklist consisting of 10 selected criteria. The result showed that only two hospitals had relatively good storage and handling practices for expired medicines. Separation of expired drugs from unexpired medicines and registration of expired medicines were both well-maintained in the facilities investigated. The lack of labeling of storage space and the storage of drugs without regard to their pharmacological groups were the two common problems encountered in all assessed hospitals. Poor management was evident in some public hospitals in the separation of expired medicines depending on their disposal method due to a lack of storage space. This pharmaceutical expiration might also lead to disruptions in healthcare delivery and poor quality of care, impeding the achievement of universal health coverage (58).

In addition to costs incurred from expired medicines, health facilities can face additional cost losses from the disposal of those medicines. In some of the studied health facilities, expired medicines were stored for more than a year past their expiration date. In contrast, the EFDA guidelines recommend that expired pharmaceuticals after expiry should not be stored for more than 6 months (55). With the exception of one hospital, the other studied hospitals disposed of their accumulated expired medicines when the prerequisites for destruction were met. In the surveyed public health institutions, open burning and landfills were the two most commonly employed techniques for disposing of obsolete pharmaceuticals. The environmental risk of pollution may result from the poor quality of landfills constructed and open burning practiced at low temperatures. The absence of appropriate disposal places was the main problem reported at the studied public health facilities. This was similar to a study conducted in Ethiopia, which stated that there was a massive accumulation of drug waste due to the absence of an appropriate disposal place (59).

The presence of expired medications in the sewage might result in an increase in antibiotic resistance to the various types of microorganisms found there, which can transform from harmless microbes into harmful and resistant pathogens (60). Due to improper disposal of outdated and expired medications, trace amounts of antibiotics have recently been found to be the cause of antibiotic resistance in humans. Non-steroidal anti-inflammatory medication (NSAID) contamination of the environment, particularly diclofenac, has been found to result in renal failure in vultures after they consume carrion from treated cattle (61). Estrogenic compounds used in oral contraceptives like 17-α-ethinylestradiol feminize fish in minute concentrations, leading to infertility (62). In addition, leftover medicines constitute another dominant cause of environmental contamination with drugs. Improper disposal of leftover medication usually contaminates the environment to a great extent (32). Drug residues from formulations like transdermal patches also leave a significant amount of drug in the environment. Transdermal patches containing fentanyl are reported to retain 28–84% of the loaded drug after removal from the skin (32). Even published literature warns that incorrect disposal of pharmaceutical wastes can cause contamination and a variety of human and animal hazards. By drinking contaminated water, people can become exposed to or accumulate traces or residues of medications from the environment (30).

The storage of expired drugs in public health facilities for more than a year resulted in significant financial losses and environmental consequences for the facilities. Expired drugs may cause financial loss to organizations for the reason of disposal, in addition to shortening of storage space. In the studied health facilities, it was reported that about 2,711.49 US$ were lost due to the disposal of those medicines for every disposal. In another country, for example, India, about 2% of their pharmaceutical budget were spent on the disposal of expired medicines (37). Shortening of storage space due to the long-term storage and disposal costs of expired drugs is another financial strain on public health facilities.

## Conclusion and recommendation

The study revealed that a total of 310,756.05 US$ was wasted in public hospitals within the Jimma Zone during the fiscal years spanning from 2019/2020 to 2020/2021. Additionally, the hospitals incur an estimated cost of 2,711.49 US$ for the disposal of expired medicines. The study revealed that the Jimma Zone public hospital medicine waste rate was above the acceptable limit of 2%. The overall waste rate of expired medicines was ~4.87%. The expiration of medicines has been linked to several issues, including near-expiry, irrational prescribing practices, and weak participation of clinicians in medicine selection and quantification of the facility. Additionally, unsatisfactory storage, handling, and disposal practices for expired medicines were observed in the public hospitals.

The lack of proper supply chain systems, policies, and program execution in Ethiopian health institutions has led to the predictable expiration of medicines. Inadequate disposal systems, inventory management, and insufficient record keeping have all contributed to the loss of drugs due to expiration. To address this issue, it is crucial for the Ethiopian Food, Medicines, and Healthcare Administration, along with the Control Authority, researchers, and healthcare professionals, to collaborate and enhance medicine procurement strategies, disposal systems, and the promotion of good storage practices. By doing so, they can effectively prevent medicine expiration in health facilities, which not only impacts the distribution of the health budget but also ecopharmacovigilance and the overall public health of the country.

## The strength of the study

The study demonstrated its robustness in addressing the identified gap and provided a comprehensive evaluation of the research question pertaining to the economic consequences of expired medications in the public hospitals of Jimma Zone. Drug expiration in public hospitals costs the distribution of health budgets and negatively affects the quality of service. The current study attempts to find out the main cause of expired medicines in public hospitals where a great number of patients are admitted. Additionally, the study found the economic impact of expired medicines and the wastage rate among the studied hospitals. The study may serve as a baseline for future findings and provide input for drug policymakers for economic analysis of the health budget.

## Limitation of the study

The study had some statistical limitations for generalizing the findings because the sampling technique used for picking the pharmacy shop manager and pharmacy store head was deliberate, which influenced the study's ultimate conclusion. The study included just a small amount of data that may or may not explain the precise number of expired medicines in the country's public hospitals. The study's findings were not compared to those of other institutions, such as the Ethiopian Pharmaceutical Supply Agency (EPSA), which had substantially better-expired pharmaceutical storage management.

## Data availability statement

The original contributions presented in the study are included in the article/Supplementary material, further inquiries can be directed to the corresponding author.

## Author contributions

YT: Methodology, Writing – original draft, Writing – review & editing. HG: Conceptualization, Data curation, Formal analysis, Investigation, Methodology, Validation, Writing – review & editing. SB: Conceptualization, Data curation, Methodology, Project administration, Validation, Writing – review & editing. GH: Conceptualization, Data curation, Methodology, Project administration, Validation, Writing – review & editing. SS: Project administration, Supervision, Writing – review & editing.
