# Supplementary material for: Assessment of the extent and monetary loss in the selected public hospitals in Jimma Zone, Ethiopia: expired medicine perspectives
Source: Front Med (Lausanne). 2024 Feb 15;11:1283070. doi: 10.3389/fmed.2024.1283070 (PMC10906092; doi:10.3389/fmed.2024.1283070)
Supplement: Supplementary file 1 [file Data_Sheet_1.docx]

# Supplementary File1. Data Collection Sheet for Medicines expiry records (2019/20 – 2020/21 G.C)

| List of hospitals | Description of Medicines Wastes  (generic & brand name, strength and dosage form) | Unit type and size | Quantity | Received budget (Year) | Expired medicines in Monetary(year) |
| --- | --- | --- | --- | --- | --- |
|  |  |  |  |  |  |
|  |  |  |  |  |  |
|  |  |  |  |  |  |
|  |  |  |  |  |  |
|  |  |  |  |  |  |

**List of public hospitals available during study period**

- 1. Jimma Medical Center
  2. Agaro General Hospitals
  3. Limu Genet General Hospitals
  4. Shenen Gibe General Hospitals
  5. Seka Chekorsa Primary Hospitals
  6. Dedo Primary Hospitals
  7. Xollay Military Hospitals (primary)
  8. Setema Primary Hospitals
  9. Nada Primary Hospitals
  10. Dimtu Primary Hospitals
  11. Jimma Military hospital (primary)
