# Supplementary material for: Assessment of the extent and monetary loss in the selected public hospitals in Jimma Zone, Ethiopia: expired medicine perspectives
Source: Front Med (Lausanne). 2024 Feb 15;11:1283070. doi: 10.3389/fmed.2024.1283070 (PMC10906092; doi:10.3389/fmed.2024.1283070)
Supplement: Supplementary file 2 [file Data_Sheet_2.docx]

**Supplementary File 2. Checklist on procedures of expired medicines storage and handling practice**

| **S.No** | **Description of Indicator** | **Yes** | **No** |
| --- | --- | --- | --- |
|  | Is there a maintained register book for recording unfit medicines? |  |  |
|  | Is there a copy of application form for past disposal of expired medicines? (observe) |  |  |
|  | Are expired medicines segregated from the usable medicines? (observe) |  |  |
|  | Expired medication are segregated based on their disposal method or are sorted into their different categories |  |  |
|  | Is room or area used for storage of expired medicines labeled properly? (“unfit for use” in red ink) |  |  |
|  | Is there a separate area to keep the expired medicines? |  |  |
|  | Is there enough storage area/space for expired medicines? |  |  |
|  | Presence of adequate security measures to avoid pilferage (e.g. Grilled gate and windows) for the area to store expired medicines. |  |  |
|  | Presence of previous disposal records (certification of destruction document). |  |  |
|  | Types of expired pharmaceuticals found in the facility (in pharmacological groups) |  |  |
