# Supplementary material for: Assessment of the extent and monetary loss in the selected public hospitals in Jimma Zone, Ethiopia: expired medicine perspectives
Source: Front Med (Lausanne). 2024 Feb 15;11:1283070. doi: 10.3389/fmed.2024.1283070 (PMC10906092; doi:10.3389/fmed.2024.1283070)
Supplement: Supplementary file 3 [file Data_Sheet_3.docx]

# Supplementary File 3: Questionnaires on storage and disposal practice of expired medication

1. **Observational-close ended questionnaire:** Managing disposal of expired pharmaceuticals by public hospitals in Jimma zone, southwest Ethiopia

Date:

1. Name of the health facility (Code )
2. Type of hospital
   1. Primary hospital
   2. General hospital
   3. Referral hospital
3. Does it happen that your medicines in the stock get expired before use?
   1. Yes b) No
4. How often do you dispose of unwanted/expired stock of pharmaceuticals?
   1. After six months
   2. After every 1 years
   3. After every 2 years
   4. When necessary (no specified time period)
5. Does your hospitals obtain approval for expired medicines disposal from appropriate regulatory body ( if yes observe necessary document)
   1. Yes b) No
6. When was your last disposal of unfit products? Mention (month and year) (See verification form or any official document to verify)
7. How much your hospital spent to dispose expired medicines in terms of monetary value ( for those who disposed previously)
8. What methods do you use regularly to dispose unfit medicines at your health facility?
   1. Landfill
   2. Incineration
   3. open burnt
   4. other (specify)
9. Does disposal certificate issued to the hospital within one weeks of disposal of expired medicines (observe the document)?
   1. Yes b) No
10. What do you think will be the dangers and problems associated with delay and improper disposal of unfit/expired pharmaceuticals?
    1. Relabeling and resold
    2. Shortage of storage space
    3. Explosion especially for explosive medicines
    4. Others (Mention)
11. What do you think is/are the barriers to proper disposal of unfit/expired pharmaceuticals?
    1. Lack of areas of disposal
    2. Long procedures of disposal
    3. High cost of disposal
    4. No barrier
    5. Others (mention)
