# Supplementary material for: Assessment of the extent and monetary loss in the selected public hospitals in Jimma Zone, Ethiopia: expired medicine perspectives
Source: Front Med (Lausanne). 2024 Feb 15;11:1283070. doi: 10.3389/fmed.2024.1283070 (PMC10906092; doi:10.3389/fmed.2024.1283070)
Supplement: Supplementary file 5 [file Data_Sheet_5.docx]

**Supplementary File 5. Perceived factors associated with medicines expiry at the public health facility**

In this section, I ask your feelings about factors contributing to medicines **Expiry** in **your facility**. For each statement on the left, please encircle one number which best describes the level of your agreement **(1 = strongly disagree; 2 = Disagree; 3 = Neutral (Neither agree nor disagree); 4 = Agree and 5 = strongly Agree)**

Questions on Perceived factors associating for Medicines expiry at the health facility

| S.  No | Associating Factors | Strongly  disagree | Disagree | Neutral | Agree | Strongly  agree |
| --- | --- | --- | --- | --- | --- | --- |
| 1. | Near expiry medicines (< 6months) are being delivered to the health facility | 1 | 2 | 3 | 4 | 5 |
| 2. | Lack of system to move nearly expired medicines from facility to facility to ensure  timely use of medicines | 1 | 2 | 3 | 4 | 5 |
| 3. | Presence of over stocked medicines due to poor quantification in the facility | 1 | 2 | 3 | 4 | 5 |
| 4. | Lack of electronic stock management tools in the health facility | 1 | 2 | 3 | 4 | 5 |
| 5. | Minimum shelf life not specified in orders | 1 | 2 | 3 | 4 | 5 |
| 6. | The shortage of pharmacy human resources in the facility | 1 | 2 | 3 | 4 | 5 |
| 7. | weak or no mechanisms for medicine expiry monitoring and evaluation in the facility | 1 | 2 | 3 | 4 | 5 |
| 8. | Poor stock management like using neither FIFO nor FEFO in stock management | 1 | 2 | 3 | 4 | 5 |
| 9. | No accurate data available in the health facility to facilitate quantification of medicines | 1 | 2 | 3 | 4 | 5 |
| 10. | Absence of functional DTC in the health facility | 1 | 2 | 3 | 4 | 5 |
| 11. | Lack of accountability for expiry of medicines in the facility | 1 | 2 | 3 | 4 | 5 |
| 12. | Medicines are purchased without procurement plan/policy in the facility | 1 | 2 | 3 | 4 | 5 |
| 13. | Selection of medicines are not based on available essential medicines list in the facility | 1 | 2 | 3 | 4 | 5 |
| 14. | Abrupt changes of treatment practices results medicines wastage in the facility | 1 | 2 | 3 | 4 | 5 |
| 15. | Medicines are not arranged systematically on shelves in the facility store | 1 | 2 | 3 | 4 | 5 |
| 16. | No time table for regular inventory level analysis | 1 | 2 | 3 | 4 | 5 |
| 17. | Weak participation of clinicians in medicine selection and quantification of the facility | 1 | 2 | 3 | 4 | 5 |
| 18. | Irrational prescribing causes underuse of certain medicines | 1 | 2 | 3 | 4 | 5 |
| 19. | Expired medicines not isolated into secure areas | 1 | 2 | 3 | 4 | 5 |
