# Supplementary material for: Assessment of the extent and monetary loss in the selected public hospitals in Jimma Zone, Ethiopia: expired medicine perspectives
Source: Front Med (Lausanne). 2024 Feb 15;11:1283070. doi: 10.3389/fmed.2024.1283070 (PMC10906092; doi:10.3389/fmed.2024.1283070)
Supplement: Supplementary file 6 [file Data_Sheet_6.docx]

## Supplementary File 6. Training manual for Data Quality Control

### [Training Manual for Data Collectors](#_Annex_III:_Data)

Trainer: **principal investigator** Trainee professionals: **pharmacy**

Number of trainers: **three** **professionals** Training Day: **two days**

Source of data: **Only public hospitals found in Jimma zone**.

**Agenda:** Training given to those data collectors on expired medicines data two-day prior data collection

**Training objective:** To give information for trainers about

- From where the data to be collected
- Types of data included and excluded
- General information about the data to be collected (name, strength, cost etc.)

**Components of training manual/trainers trained on the following issues**

- The data to be gathered was from only expired revolving drug fund
- Medicines that had expired in years of **2019/20-2020/21** were included for volume and cost analysis
- Samples from program and anticancer drugs were excluded
- Expired laboratory reagents and supplies data were excluded
- Samples that have been reported as damaged was excluded
- Samples that did not reported with their cost was checked and asked for their purchased value in respective public hospitals
- The detailed information on expired medicines collected was trained based on the table below (annex III: b)

**Note:** This Training manual was developed using Ethiopian pharmaceuticals waste disposal guidelines 2011 E.C, and peer reviewed published literature.
